# Ursolic acid inhibits the invasive phenotype of SNU-484 human gastric cancer cells

**DOI:** 10.3892/ol.2014.2735

**Published:** 2014-11-24

**Authors:** EUN-SOOK KIM, AREE MOON

**Affiliations:** College of Pharmacy, Innovative Drug Center, Duksung Women’s University, Seoul 132-714, Republic of Korea

**Keywords:** ursolic acid, cell invasion, matrix metalloproteinase

## Abstract

Metastasis is a major cause of cancer-related mortality in patients with gastric cancer. Ursolic acid, a pentacyclic triterpenoid compound derived from medicinal herbs, has been demonstrated to exert anticancer effects in various cancer cell systems. However, to the best of our knowledge, the inhibitory effect of ursolic acid on the invasive phenotype of gastric cancer cells has yet to be reported. Therefore, the aim of the present study was to investigate the effect of ursolic acid on the invasiveness of SNU-484 human gastric cancer cells. Ursolic acid efficiently induced apoptosis, possibly via the downregulation of B-cell lymphoma 2 (Bcl-2), the upregulation of Bcl-2-associated X protein and the proteolytic activation of caspase-3. Furthermore, the activation of p38 mitogen-activated protein kinase and c-Jun N-terminal kinase was increased by the administration of ursolic acid. In addition, ursolic acid significantly suppressed the invasive phenotype of the SNU-484 cells and significantly decreased the expression of matrix metalloproteinase (MMP)-2, indicating that MMP-2 may be responsible for the anti-invasive activity of ursolic acid. Taken together, the results of the present study demonstrate that ursolic acid induces apoptosis and inhibits the invasive phenotype of gastric cancer cells; therefore, ursolic acid may have a potential application as a chemopreventive agent to prevent the metastasis of gastric cancer or to alleviate the process of metastasis.

## Introduction

Gastric cancer is the second leading cause of cancer-related mortality worldwide (1) and the predominant cause of cancer-related mortality in numerous Asian countries, including South Korea and China (2). Gastric cancer is a multifactorial disease involving genetic and environmental factors (3). Furthermore, the prognosis of advanced gastric cancer patients remains poor due to the high rate of metastatic recurrence (4,5).

Ursolic acid is a pentacyclic triterpenoid compound that is derived from a range of medicinal herbs, including *Rosemarinus officinalis*, *Eribotrya japonica*, *Calluna vulgaris* and *Oldenlandia diffusa* (6–8). Ursolic acid has been demonstrated to exert a number of anticancer activities, including the inhibition of tumorigenesis, tumor promotion and angiogenesis (7), as well as the induction of apoptosis in various cancer cell lines, including melanoma, breast, gastric and hepatocellular carcinoma, human non-small cell lung cancer and liver cancer cell lines (7–12). In addition, ursolic acid has been reported to inhibit *in vivo* tumor growth in various animal models (13–15), and to suppress invasion and migration in human lung, breast and ovarian cancer cells (10,16,17). However, to the best of our knowledge, the anti-invasive activity of ursolic acid in gastric cancer cells has yet to be reported. Therefore, the present study aimed to investigate the inhibitory effect of ursolic acid on the growth and invasive phenotype of SNU-484 human gastric cancer cells.

The process of tumor metastasis occurs by tumor cells disseminating from the primary tumor to distant secondary organs or tissues. Metastasis involves multiple steps, including the invasion, migration and dissemination of malignant tumor cells (18). Furthermore, cancer cell invasion has been extensively associated with the increased expression of matrix-degrading matrix metalloproteinase (MMP) enzymes (19,20).

Apoptosis involves a series of cellular events that results in the activation of apoptosis-associated gene products, such as B-cell lymphoma 2 (Bcl-2), Bcl-2-associated X protein (Bax) and the caspases (21,22). The Bcl-2 and Bax proteins are functionally opposed: Whereas Bcl-2 acts to inhibit apoptosis, Bax promotes apoptosis (23,24). Caspase-3 is an apoptosis-associated cysteine peptidase that interacts with caspase-8 and -9, and is key in mammalian cell apoptosis (25).

In addition, a number of other molecules are involved in the process of apoptosis. The mitogen-activated protein kinase (MAPK) signaling pathways modulate gene expression, proliferation, motility and apoptosis (26–29). Activation of c-Jun N-terminal kinase (JNK) and p38 MAPK have been demonstrated to trigger apoptosis (27,30,31), and extracellular signal-regulated kinase (ERK) activity promotes apoptotic pathways via the induction of mitochondrial cytochrome *c* release, caspase-8 activation or permanent cell cycle arrest (32).

In order to examine the chemopreventive potential of ursolic acid in gastric cancer cells *in vitro*, the present study evaluated the effects of ursolic acid on proliferation, apoptosis and the invasive phenotype of SNU-484 human gastric cancer cells.

## Materials and methods

### Reagents

Ursolic acid (3β-hydroxy-urs-12-en-28-oic acid), dimethyl sulfoxide (DMSO) and MTT were purchased from Sigma-Aldrich (St. Louis, MO, USA). Dulbecco’s modified Eagle’s medium (DMEM) was purchased from Cellgro Mediatech (Manassas, VA, USA), and fetal bovine serum and penicillin-streptomycin were purchased from Invitrogen Life Technologies (Grand Island, NY, USA). The annexin V-fluorescein isothiocyanate (FITC) apoptosis detection kit I was purchased from BD Biosciences (San Diego, CA, USA).

### Cell lines

The SNU-484 gastric cancer cell line was provided by Dr H.D. Um (Korean Institute of Radiological and Medical Science, Seoul, South Korea). The SNU-484 cells were cultured in DMEM supplemented with 10% heat-inactivated fetal bovine serum and 100 U/ml penicillin-streptomycin. The cells were maintained in a humidified atmosphere of 95% air and 5% CO_2_ at 37°C.

### MTT assay

SNU-484 cells cultured in a 96-well plate were treated with various concentrations of ursolic acid (0, 1, 5, 10 and 25 μM) for 24 h. Control cells were treated with DMSO equal to the highest percentage of solvent used under experimental conditions. Briefly, 25 mg/ml 0.5% MTT was added to the media and the cells were incubated for an additional 4 h. Following removal of 100 μl supernatant, which was replaced with an equal volume of DMSO, the absorbance was measured at a wavelength of 540 nm using an enzyme-linked immunosorbent assay (ELISA) plate reader (ELx800™; BioTek Instruments, Inc., Winooski, VT, USA). The percentage of surviving cells was defined as the relative absorbance of treated versus untreated cells.

### Flow cytometry analysis

The cells were grown in six-well plates and incubated for 24 h prior to treatment with ursolic acid. After 24 h, the cells were harvested and washed twice with phosphate-buffered saline (PBS). The cells (5×10^5^) were subsequently double-stained with FITC-conjugated annexin V and propidium iodide for 15 min at room temperature in 1X binding buffer, followed by analysis using a Vision CBA Image Cytometry system (Nexcelom Bioscience LLC, Lawrence, MA, USA).

### Immunoblot analysis

The cells were cultured to 70% confluency and incubated in serum-free media containing various concentrations of ursolic acid (0.0, 1.0, 2.5 and 5.0 μM) for 24 h. To prepare whole-cell extracts, the cells were washed with PBS and lysed by adding SDS-lysis buffer containing protease inhibitor cocktail (Roche Diagnostics GmbH, Mannheim, Germany). Equal amounts of protein were subjected to 12% SDS-PAGE analysis and electrophoretically transferred to a polyvinylidene fluoride membrane (Bio-Rad Laboratories, Hercules, CA, USA). Subsequently, the membranes were blocked with 5% w/v skimmed dried milk in PBS with Tween-20 (PBST) and incubated with primary (dilution, 1:1,000 in PBST) and secondary (dilution, 1:4,000 in PBST) antibodies, and detected using an enhanced chemiluminescence western blotting detection system (GE Healthcare Life Sciences, Chalfont, UK). Rabbit polyclonal anti-human p38 MAPK, rabbit polyclonal anti-human phosphorylated (phospho) p38 MAPK, rabit polyclonal anti-human phospho ERK1/2, mouse monoclonal anti-human ERK1/2, rabbit polyclonal anti-human JNK, mouse monoclonal anti-human phospho JNK and rabbit polyclonal anti-human poly(ADP-ribose) polymerase (PARP) antibodies were purchased from Cell Signaling Technology, Inc. (Beverly, MA, USA). Mouse monoclonal anti-human Bcl-2, mouse monoclonal anti-human caspase-3 and rabbit polyclonal anti-human Bax antibodies were purchased from Santa Cruz Biotechnology, Inc. (Santa Cruz, CA, USA).

### In vitro invasion assays

An *in vitro* invasion assay was performed using a 24-well Transwell^®^ unit with polycarbonate filters (Corning Costar, Inc., Cambridge, MA, USA). The lower side of the filter was coated with type I collagen, the upper side was coated with Matrigel (Collaborative Research, Lexington, KY, USA), the upper compartment of the Transwell^®^ unit was filled with serum-free medium and the lower compartment was filled with the culture medium. Ursolic acid (0.0, 1.0, 2.5 and 5.0 μM) was also added to each compartment. The cells were placed on the upper side of the Transwell^®^ plate and incubated for 24 h. The cells that remained on the upper surface of the filter were removed with a cotton swab, while the cells that had migrated through the filter to the lower compartment were fixed with methanol and stained with crystal violet for 20 min. The crystal violet dye retained on the filters was extracted using 30% acetic acid and cell invasion was measured by reading the absorbance at 595 nm using an ELISA plate reader (ELx800; Bio-Tek Instruments).

### Gelatin zymography assay

The cells were cultured to 70% confluency and incubated in serum-free media containing various concentrations of ursolic acid (0.0, 1.0, 2.5 and 5.0 μM) for 48 h. The conditioned medium was collected and centrifuged at 13,000 × g for 10 min to remove cell debris. Subsequently, the protein concentration was measured using bicinchoninic acid assay reagents (Pierce Biotechnology, Inc., Rockford, IL, USA), and equal amounts of protein from the conditioned media were electrophoresed on 10% SDS-PAGE gels containing 1 mg/ml gelatin. Following electrophoresis, the gels were washed with renaturation buffer (2.5% Triton X-100) three times for 30 min, rinsed for 15 min with developing buffer [50 mM Tris-HCl buffer (pH 7.6) containing 5 mM CaCl_2_, 0.02% Brij-35 and 0.2% sodium azide], and incubated overnight at 37°C. The gels were stained with staining buffer (0.5% Coomassie Brilliant Blue R-250 solution containing 10% acetic acid and 20% methanol) for 30 min and destained with 10% acetic acid solution. Areas of gelatinase activity were detected as clear bands against the blue-stained gelatin background, and relative band intensities were determined by the quantification of each band using the Gel Doc™ XR+ imaging system (Bio-Rad Laboratories).

### Statistical analysis

The results are presented as the mean ± standard deviation of three independent experiments run in triplicate and analyzed by Student’s t-test. P<0.05 was considered to indicate a statistically significant difference.

## Results

### Ursolic acid inhibits cell growth in SNU-484 cells

**Figure 1:**
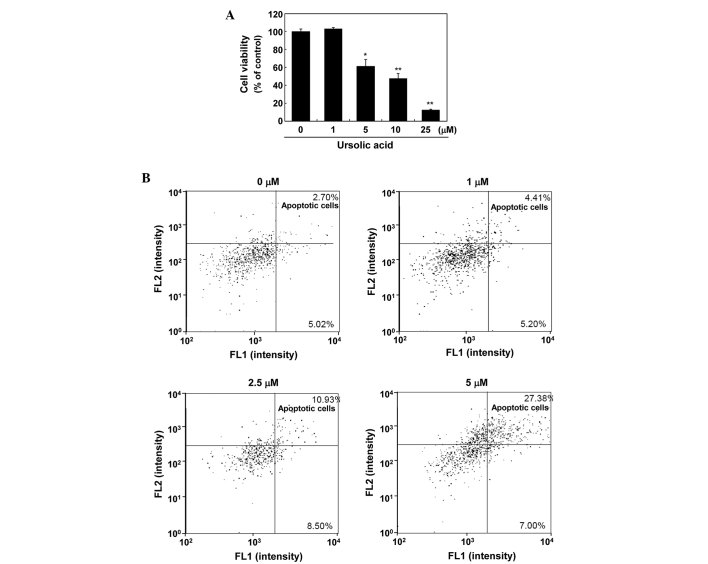
Ursolic acid inhibits the growth of SNU-484 cells. (A) The cells were treated with various concentrations of ursolic acid for 24 h and then subjected to an MTT assay. The results represent the mean ± standard error of the mean for triplicates. ^*^P<0.05 and ^**^P<0.01 vs. control. (B) The cells were treated with various concentrations of ursolic acid (0, 1.0, 2.5 and 5.0 μM) for 24 h and incubated with annexin V-fluorescein isothiocyanate (FITC) and propidium iodide (PI). The FITC and PI fluorescence was measured using a Vision CBA Image Cytometry system with FL-1 (530 nm) and FL-2 (585 nm) filters, respectively.

To investigate the effect of ursolic acid on the growth of SNU-484 gastric cancer cells, an MTT assay was performed on cells treated with various concentrations of ursolic acid. As demonstrated in Fig. 1A, treatment of the SNU-484 cells with ursolic acid for 24 h inhibited growth in a dose-dependent manner (P<0.01), with a half maximal inhibitory concentration of 9 μM.

### Ursolic acid induces apoptosis and regulates the expression of apoptosis-associated proteins

To investigate whether ursolic acid-induced growth inhibition involves apoptosis, a flow cytometric analysis was conducted. As demonstrated in Fig. 1B, ursolic acid induced the apoptosis of the SNU-484 cells in a concentration-dependent manner. Treatment of the SNU-484 cells with 5 μM ursolic acid for 24 h resulted in a significant increase in annexin V- and propidium iodide -positive apoptotic cells (27.38%) compared with the control cells (2.70%). These results indicate that the observed growth inhibitory effect of ursolic acid may be due to the induction of apoptotic cell death.

**Figure 2:**
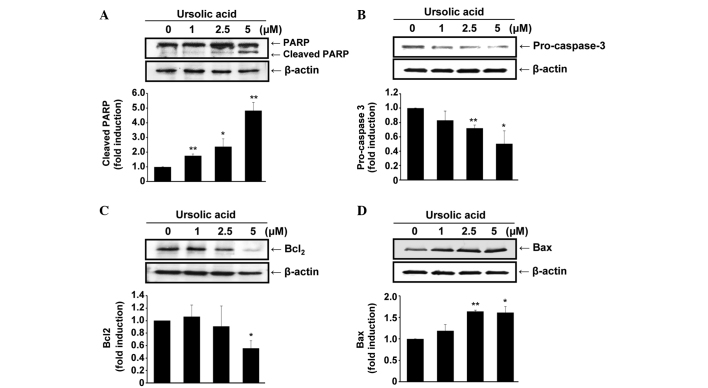
Ursolic acid regulates the expression of apoptosis-associated proteins in SNU-484 cells. Cells were treated with various concentrations of ursolic acid (0, 1.0, 2.5 and 5.0 μM) for 24 h and the protein expression levels of levels of (A) PARP, (B) pro-caspase 3, (C) Bcl-2 and (D) Bax were determined by performing an immunoblot analysis. ^*^P<0.05 and ^**^P<0.01 vs. control. PARP, poly(ADP-ribose) polymerase; Bcl-2, B-cell lymphoma 2; Bax, Bcl-2-associated X protein.

To evaluate the molecular mechanisms underlying ursolic acid-induced apoptosis, the protein expression levels of PARP, pro-caspase-3, Bcl-2 and Bax were determined in cells treated with ursolic acid for 24 h. PARP, a well-established substrate for caspase-3 and thus an indicator of apoptosis (33), appeared to be cleaved by ursolic acid (P<0.01; Fig. 2A), and the expression of pro-caspase-3 was decreased by ursolic acid (P<0.05; Fig. 2B), indicating that ursolic acid may induce the activation of caspase-3 in the SNU-484 cells.

In addition, the expression levels of Bcl-2 and Bax were examined in the ursolic acid-treated cells. Ursolic acid dose-dependently inhibited the expression of Bcl-2 (P<0.05), whereas Bax expression levels were increased by the administration of ursolic acid (P<0.05; Fig. 2C and D). These data demonstrate that ursolic acid induces apoptosis in SNU-484 cells, possibly by downregulating the anti-apoptotic Bcl-2 protein and upregulating the pro-apoptotic Bax protein.

### Ursolic acid activates p38 MAPK and JNK

**Figure 3:**
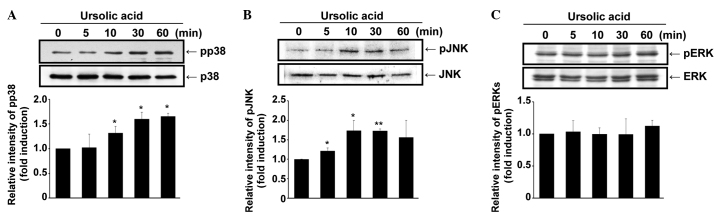
Ursolic acid activates p38 mitogen-activated protein kinase (MAPK) and JNK in SNU-484 cells. The cells were treated with 5 μM ursolic acid at the indicated time-points. The levels of phosphorylated and total (A) p38 MAPK, (B) JNK and (C) ERK were determined by performing immunoblot analysis. ^*^P<0.05 and ^**^P<0.01 vs. control. JNK, c-Jun N-terminal kinase; p, phosphorylated; ERK, extracellular signal-regulated kinase.

A kinetic study was performed to examine the effect of ursolic acid on the activation of MAPKs. As demonstrated in Fig. 3A, phospho-p38 MAPK expression was increased in the SNU-484 gastric cancer cells by the administration of ursolic acid in a time-dependent manner (P<0.05). In addition, the phosphorylation level of JNK increased up to 10 min after ursolic acid treatment (P<0.01) and then slightly decreased up to 60 min after (Fig. 3B). By contrast, the activation of ERK and phospho-ERK was not significantly altered by the administration of ursolic acid (Fig. 3C). These results demonstrate that ursolic acid activates p38 MAPK and JNK, indicating the possible involvement of these signaling pathways in the ursolic acid-induced apoptosis of SNU-484 gastric cancer cells.

### Ursolic acid inhibits the invasive phenotype of SNU484 cells

**Figure 4:**
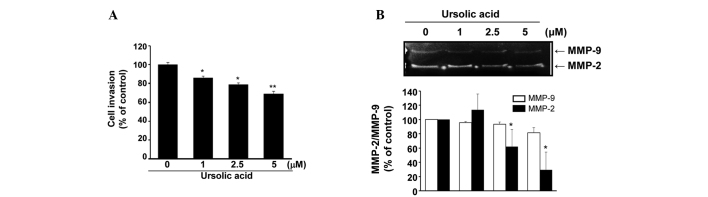
Ursolic acid inhibits invasion and MMP-2 expression in SNU-484 cells. (A) An *in vitro* invasion assay was conducted on SNU-484 cells treated with various concentrations of ursolic acid (0, 1.0, 2.5 and 5.0 μM) for 24 h and a (B) gelatin zymogram assay was conducted on conditioned media of SNU-484 cells treated with ursolic acid for 24 h. ^*^P<0.05 and ^**^P<0.01 vs. control. MMP, matrix metalloproteinase.

To examine the effect of ursolic acid on the invasive phenotype of the SNU-484 cells, an *in vitro* invasion assay was performed. As demonstrated in Fig. 4A, the invasive ability of the SNU-484 cells was significantly inhibited by ursolic acid administration in a dose-dependent manner (P<0.01). In addition, the effect of ursolic acid on MMP-2 and/or MMP-9 in the SNU-484 cells was examined. As demonstrated in Fig. 4B, the expression level of MMP-2 was dose-dependently decreased by the administration of ursolic acid (P<0.05), while the expression of MMP-9 was not significantly reduced. These data indicate that ursolic acid may inhibit the invasiveness of SNU-484 cells via the downregulation of MMP-2.

## Discussion

Gastric cancer is one of the predominant human malignancies, and chemoprevention using natural products has previously been considered as a promising approach for the control and management of cancer (34,35). The present study demonstrated that ursolic acid efficiently induces apoptosis and inhibits the invasive phenotype of SNU-484 human gastric cancer cells. These findings indicate a potential application of ursolic acid in regulating the aggressiveness of human gastric cancer.

Cancer cells often escape apoptosis by increasing the expression levels of anti-apoptotic genes or by decreasing the expression of pro-apoptotic genes (22). Ursolic acid has previously been demonstrated to induce apoptosis by causing the release of cytosolic cytochrome *c*, activating caspase-3, reducing the expression of Bcl-2 and increasing the expression of Bax in HeLa (36) and MDA-MB-231 breast cancer cells (37). Similarly, the results of the present study indicate that ursolic acid may induce apoptosis by decreasing the expression of Bcl-2 and increasing the expression of Bax in SNU-484 gastric cancer cells.

The MAPK family of proteins regulate various stress responses, and thus are crucial for the maintenance of cells (38). Among the various MAPK pathways, the JNK and p38 MAPK pathways have been indicated to be key regulators of stress-induced apoptosis (39). There has previously been controversy regarding the role of p38 MAPK signaling in cell death; depending on the cell system used, p38 MAPK signaling has been shown to promote cell death (40,41), and to enhance cell survival and growth (42,43). Furthermore, ursolic acid has been demonstrated to induce apoptosis via the activation of JNK, but not p38 MAPK, in pituitary adenoma, human leukemia K562 and prostate cancer cells (42–46). The results of the present study demonstrated that ursolic acid activates JNK and p38 MAPK in human gastric cancer cells, indicating that the role of MAPK signaling in the induction of apoptosis may be cell-type specific.

Tumor cell invasion is an early step of the metastatic cascade, representing the onset of the transition from the benign stage to malignant progression (47). Numerous studies have identified an association between cell invasion and the inhibition of MMPs (48–50). Furthermore, our previous study and another study have demonstrated that among the MMPs, MMP-2 expression is associated with the invasive phenotype of gastric cancer cells (51,52). Consistent with these previous studies, the present study demonstrated that ursolic acid inhibits the invasive phenotype of SNU-484 gastric cancer cells by downregulating MMP-2 expression.

In conclusion, the present study clearly demonstrates that ursolic acid induces apoptosis and inhibits cell invasion in SNU-484 human gastric cancer cells. Considering that gastric cancer is one of the most common types of cancer and that metastasis is the major cause of mortality in gastric cancer patients, the findings of the present study may provide insights into the potential application of ursolic acid as an agent for the prevention and treatment of human gastric cancer.
